# SSU rRNA sequencing data for bacterial communities associated with *Vibrio-*infected rainbow trout (*Oncorhynchus mykiss)*

**DOI:** 10.1016/j.dib.2022.108752

**Published:** 2022-11-13

**Authors:** Aleksey Morozov, Alina Vasileva, Natalia Chechkova, Tamara Kuchko, Ekaterina Borvinskaya, Irina Sukhovskaya

**Affiliations:** a Limnological Institute SB RAS, 3, Ulan-Batorskaya, Irkutsk 664033, Russia; b Petrozavodsk StateUniversity, 33, Lenina st., Petrozavodsk 185910, Russia; c Irkutsk State University,1, Karla Marksa st., Irkutsk 664003, Russia; d Institute of Biology of Karelian Research Centre RAS, 11, Pushkinskayast., Petrozavodsk 185910, Russia

**Keywords:** Trout, Vibriosis, SSU rRNA, Metabarcoding, Gut bacteria, Spleen, Opportunistic infection

## Abstract

*Vibrio anguillarum* infection in aquacultured trout, besides its own harmful effects, can also make the fish more susceptible to other infections, both by swamping the host's immune system and by creating the skin lesions that serve as a direct gateway for opportunistic bacteria. Tissue samples were taken from the intestines, spleen and skin lesions of cage-grown rainbow trout suffering from natural *Vibrio* infections of varying severity. In addition, a water sample was taken to document the planktonic bacteria inhabiting the fish farm at the time of the study. Total DNA was isolated from these samples and used to produce v3-v4 SSU rRNA amplicons, which were then sequenced on Illumina MiSeq (2 × 300 bp) and assembled into 97% identity OTUs. This dataset allows analysis of bacteria co-colonizing the skin and intestines of infected fish along with the pathogenic *Vibrio anguillarum*, as well as provides information on the spread of bacteria between various bodily compartments of trout.

**Specifications Table**

**Table:** 

Subject	Aquaculture, Applied Microbiology
Specific subject area	Analysis of bacterial communities associated with cultured fish infected with *Vibrio anguillarum*
Type of data	*Sequencing data deposited at NCBI SRA* *Tables deposited at Mendeley Data*
How the data were acquired	Amplicon high-throughput sequencing on Illumina MiSeq followed with Mothur analysis to produce OTU tables and taxonomies.
Data format	Raw Analyzed
Description of data collection	Gut (the pooled contents of the anterior and posterior intestines) and spleen samples were taken under aseptic conditions from 19 cage-grown trout specimens. Skin mucus samples were taken from 10 fish with skin lesions. A surface water sample was taken near a cage at a fish farm. Total DNA was isolated and V3-V4 SSU rRNA fragments were amplified and sequenced using standard protocols. Analysis was performed in Mothur following MiSeq SOP with SILVA SSU rRNA reference alignment.
Data source location	Institute of Biology, Ecology and Agricultural Technologies, Petrozavodsk State University, Petrozavodsk, Russia. Rainbow trout, *O. mykiss,* was obtained in August 2020 from a commercial trout farm located on the White Sea, Murmansk region, Russia.
Data accessibility	**Raw sequencing data** Repository name: NCBI Short Read Archive Data identification number: PRJNA828372 Direct URL to data: https://www.ncbi.nlm.nih.gov/bioproject/PRJNA828372 **OTU table and OTU taxonomy** Repository name: Mendeley Data Data identification number: 6m4jz3ywhc Direct URL to data: https://data.mendeley.com/datasets/6m4jz3ywhc/3

## Value of the Data

• The present dataset for the first time provides insight into the co-infection of cultured rainbow trout with opportunistic bacteria during *Vibrio* infection, which allows association of microbiota composition with pathological status.
• The dataset provides insight on the reorganization of the gut and skin microbiota upon infection with vibrios, which may play a role in the impairment of the barrier functions of these organs.
• These data may be useful in expanding knowledge of the dissemination of Vibrio and other bacteria in the blood and body compartments of infected fish.
• For fish farmers and aquaculture researchers, these data can be used as a reference to identify possible infection.
• The data contribute to monitoring the global distribution of virulent Vibrio strains in natural marine and brackish waters.

## 1 Objective

This dataset was generated to document the bacterial community of rainbow trout (*Oncorhynchus mykiss*) suffering from vibriosis, a common disease in cultured fish and a significant threat to aquaculture. Although this community is dominated by the pathogenic *Vibrio anguillarum*, other bacteria may also affect the development of vibriosis. Since none of the existing metabarcoding studies of cultured trout were performed under vibriosis condition, nor have the bacteria co-infecting trout along with *Vibrio sp* been thoroughly studied by classical methods, there is very little known about the composition of these bacteria. Sequencing was performed on samples from multiple body compartments to study the spread of *Vibrio* and other bacteria within infected specimen. Further, the experiment was designed to include fish with varying vibriosis severity, which allows searching for the correlations between this severity and composition of bacteria in various trout-associated communities.

## 2 Data Description

The obtained dataset consists of raw sequencing data deposited at NCBI SRA [dataset] [1] and OTU tables deposited at Mendeley Data [dataset] [2] (table “OTUs'' contains un-normalized abundances of each OTU and sample descriptions; table “Taxonomy”contains these OTUs’ taxonomy for ranks from domain to genus). Sample metadata (specimen ID, specimen status and organ sampled) are provided both at NCBI (Biosample IDs SAMN27652302 to SAMN27652349) and as columns in OTU table.

**Fig. 1:**
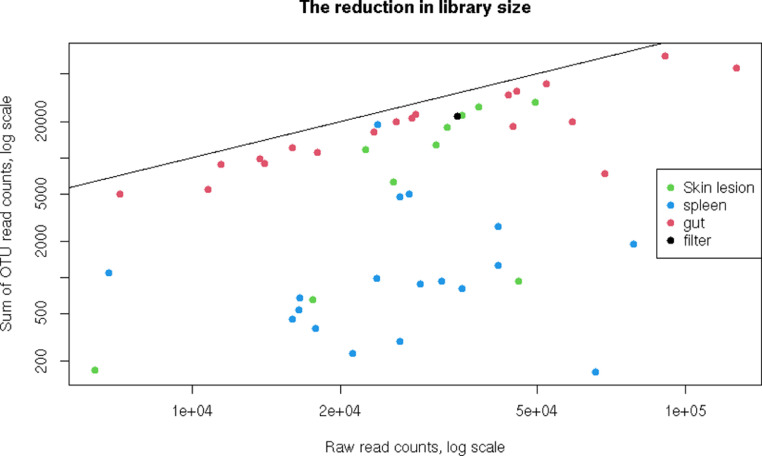
The scatter plot of the total abundance of final OTUs (number or reads after filtration) against raw library size before filtration. The black line corresponds to 1:1 ratio, i.e. no excluded reads.

The raw sequencing data were published as is, without any processing or filtering other than adapter trimming and demultiplexing. OTUs, on the other hand, were built only from reads that passed quality control (including read pair assembly, filtering by length and quality, and chimera removal) and correctly aligned to the SILVA SSU rRNA reference alignment. Singleton OTUs, defined as those that did not include more than one read in at least one library, have also been excluded. In some libraries, this results in a significant decrease in the number of reads (Fig. 1).

**Fig. 2:**
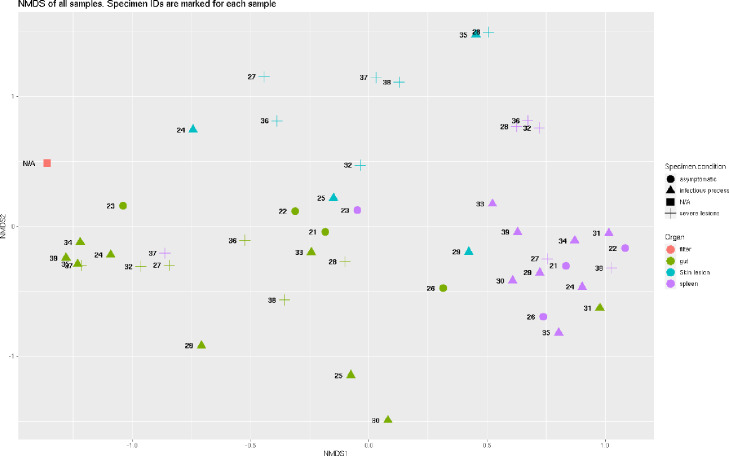
Overall similarity of produced libraries, as revealed by NMDS. The shape of the marker denotes the physiological condition of the specimen, while the color denotes the organs. Specimen ID is marked for each sample (N/A in case of water sample).

**Fig. 3:**
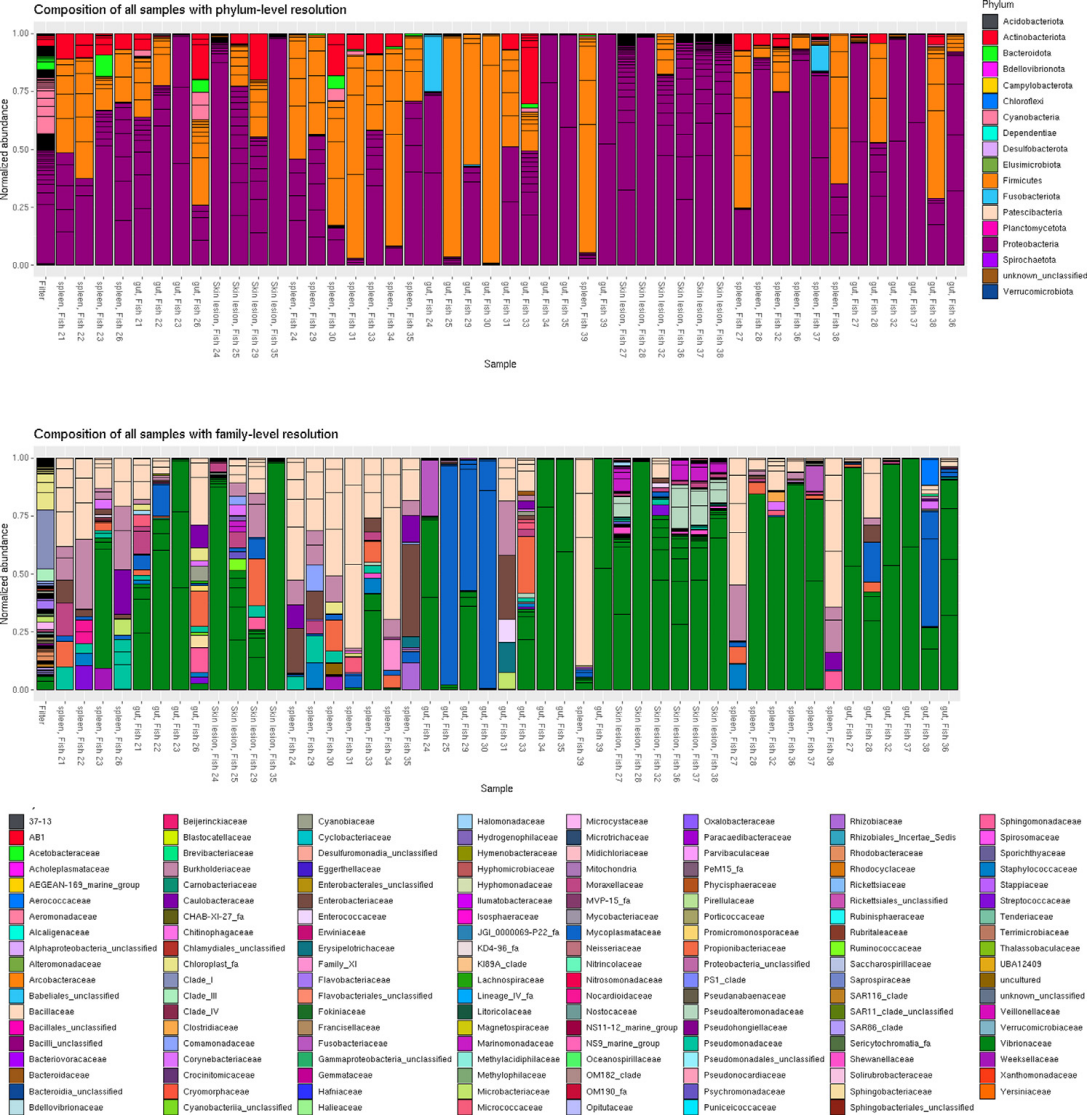
The taxonomic composition of the produced libraries.

Overall similarity of the libraries is shown via NMDS using Bray-Curtis distance at Fig. 2; taxonomic composition with phylum- and genus-level resolution is shown at Fig. 3. All plots were built for normalized abundances.

## 3 Experimental Design, Materials and Methods

The fish was caught in August 2020 at a trout farm located in the bay of the White Sea in the North-West of Russia. Fish were caught with a net in a random order from four neighboring cages. Individuals were caught with obvious signs of infection and not showing external signs of the disease. A total of 19 fish were sampled, of which 4 were asymptomatic, 9 moderately infected and 6 severely injured. The physiological state of fish specimens was estimated according to a number of parameters, documented in [3].

Each fish was euthanized in a 0.5 ml/L clove oil emulsion for about 5 min. In the presence of skin lesions, the mucus was scraped off the affected area with a sterile scalpel and fixed in liquid nitrogen. Then the fish were aseptically dissected, the spleen of the fish was weighed and fixed in liquid nitrogen. The intestines were opened with scissors, the contents and parietal microflora of the anterior and posterior intestines were scraped off with a sterile scalpel, pooled, and fixed in liquid nitrogen. Water samples with a volume of 10 L were taken near the cage and filtered through a sterile 0.22 µm filter cartridge. The filter was then frozen and stored in liquid nitrogen until DNA extraction.

Total DNA was isolated from samples using a DiaGene kit for DNA isolation (Dia-M, Russia). For this, 50 mg of the sample was homogenized in lysis LBT buffer with the addition of 8 μl of proteinase K and incubated for 16 hours at 37°C. The obtained lysate was centrifuged in an Eppendorf centrifuge at 12000 g for 20 minutes. A sorption buffer (1:1.4 v/v) and chloroform (1:1 v/v) were added to the supernatant and centrifuged at 12000 g for 20 minutes. Then, the aqueous phase containing DNA was collected and transferred into DiaGene microcolumns (Dia-M, Russia). The columns were washed once with WB1 buffer and twice with WB2 buffer. DNA from the columns was eluted with sterile deionized water.

The quality of the obtained genomic DNA samples was tested by nucleic acid electrophoresis in an agarose gel. V3–V4 variable regions of the 16S rRNA gene were amplified using the "16S Metagenomic Sequencing Library Preparation" protocol (Part # 15044223 Rev. B; Illumina), with the primer pair 5′ TCGTCGGCAGCGTCAGATGTGTATAAGAGACAGCCTACGGGNGGCWGCAG and 5′ GTCTCGTGGGCTCGGAGATGTGTATAAGAGACAGGACTACHVGGGTATCTAATCC. After obtaining the amplicons, the libraries were cleaned and mixed equimolarly using a SequalPrep™ Normalization Plate Kit (ThermoFisher, Cat # A10510-01). The resulting pool of libraries was tested by capillary electrophoresis and sequenced on an Illumina MiSeq (2 × 300 bp paired-end reads) with a MiSeq Reagent Kit v3 (600 cycles). The PhiX phage library was used to control the sequencing parameters. Most of the reads related to phage DNA were removed during the demultiplexing process.

The obtained paired-end Illumina MiSeq reads were assembled into contigs. After deleting the low-quality reads, unassembled pairs (those that produce contigs above 470 nucleotides in length) and chimeric reads, contigs were mapped to SILVA v138.1 reference SSU rRNA alignment [4]. Sequences that aligned correctly were used to generate 97% identity OTUs. All read analyses were performed in mothur 1.44.11 software [5]. OTUs that have no more than one read in all libraries were excluded as singletons. All plots were built in R v.4.1.2 using the phyloseq library [6].

## Ethics Statements

All animal handling procedures were approved by the Institutional Review Board (or Ethics Committee) of Petrozavodsk State University (protocol code №1 20/05/2020), following EU-established norms and procedures.

## CRediT Author Statement

**Irina Sukhovskaya** and **Ekaterina Borvinskaya:**Conceptualization; **Irina Sukhovskaya** and **Tamara Kuchko:** Project administration; **Irina Sukhovskaya** and **Ekaterina Borvinskaya:** Supervision; **Aleksey Morozov, AP, IM, Alina Vasileva, Natalia Chechkova** and **Tamara Kuchko:** Methodology; **Aleksey Morozov** and **Ekaterina Borvinskaya:** Writing – original draft; **Irina Sukhovskaya, Aleksey Morozov** and **AP:** Writing – review & editing. All authors contributed to the article and approved the submitted version.

## Declaration of Competing Interest

The authors declare that they have no known competing financial interests or personal relationships that could have appeared to influence the work reported in this paper.

## Data Availability

Composition of bacterial communities in the intestine, skin and spleen of farmed rainbow trout infected with Vibrio anguillarum (Original data) (Mendeley Data). SSU rRNA sequencing of various rainbow trout organs under bacterial infection (Original data) (NCBI).

## References

[1] A. A. Morozov, SSU rRNA sequencing of various rainbow trout organs under bacterial infection. NCBI sequence read archive, 2022. https://www.ncbi.nlm.nih.gov/bioproject/PRJNA828372 [dataset]

[2] Morozov A., Sukhovskaya I., Chechkova N., Borvinskaya E., Vasileva A. (2022). Composition of bacterial communities in the intestine, skin and spleen of farmed rainbow trout infected with Vibrio anguillarum. Mendeley Data.

[3] Kurpe S.R., Sukhovskaya I.V., Borvinskaya E.V., Morozov A.A., Parshukov A.N., Malysheva I.E., Vasileva A.V., Chechkova N.A., Kuchko T.Y. (2022). Physiological and biochemical characteristics of rainbow trout with severe, moderate and asymptomatic course of *Vibrio anguillarum* infection. MDPI Anim..

[4] Pruesse E., Quast C., Knittel K., Fuchs B.M., Ludwig W., Peplies J., Glöckner F.O. (2007). SILVA: a comprehensive online resource for quality checked and aligned ribosomal RNA sequence data compatible with ARB. Nucleic Acids Res..

[5] Schloss P.D., Westcott S.L., Ryabin T., Hall J.R., Hartmann M., Hollister E.B., Lesniewski R.A., Oakley B.B., Parks D.H., Robinson C.J., Sahl J.W., Stres B., Thallinger G.G., van Horn D.J., Weber C.F. (2009). Introducing mothur: open-source, platform-independent, community-supported software for describing and comparing microbial communities. Appl. Environ. Microbiol..

[6] McMurdie P.J., Holmes S. (2013). phyloseq: an R package for reproducible interactive analysis and graphics of microbiome census data. PloS One.

